# Integrating text analytics and statistical modelling to analyse kidney transplant immune suppression medication in registry data

**DOI:** 10.23889/ijpds.v1i1.353

**Published:** 2017-04-19

**Authors:** Ghada Alfattni, Niels Peek, Goran Nenadic, Fergus Caskey

**Affiliations:** 1 School of Computer Science, University of Manchester; 2 Health eResearch Centre, University of Manchester; 3 UK Renal Registry

## Objectives

Electronic Health Records (EHRs) contain a wealth of routinely-collected data that could potentially be used to inform clinical decisions such as the choice between competing treatment regimens. Apart from structured data about diagnoses and biomarkers, these records often include unstructured data such as free-text medication prescriptions. Disjoint toolsets exist for structured and unstructured data, making it difficult to analyse datasets that comprise both structured and unstructured data. Representing free-text items in a structured and standardised format would enable their statistical analysis. The aim of this study was to develop a generic, analytical pipeline that integrates different tools for text analytics and statistical modelling, and to apply it to data from the UK Renal Registry (UKRR) to answer specific clinical and epidemiological questions on kidney transplant immune suppression.

## Approach

The UKRR database comprises data from all renal units in England, Wales and Northern Ireland and consists of both structured and semi-structured data. Our workflow starts by using rules to extract medication regimen from free-text prescriptions, which is then automatically combined with structured patient's record. The data is then analysed for patterns of transplant immune suppression prescribing by specific centres across the UK.

## Results

We have developed an analytical pipeline for improving concordance between unstructured and structured medical records, combining new and established text analysis and subsequence analysis tools. Results of the study are underway and will be presented at the conference.

## Conclusion

We have developed new framework that integrates tools for text analytics and statistical modelling, to facilitate the analysis of mixed structured and unstructured data. Our analysis of the UKRR data will help to compare immune suppressive treatment regimens, identify best practice, and explore the associations between transplant medication and transplant outcomes.

